# The Effect of the Crosstalk between Photoperiod and Temperature on the Heading-Date in Rice

**DOI:** 10.1371/journal.pone.0005891

**Published:** 2009-06-12

**Authors:** Weijiang Luan, Huizhe Chen, Yaping Fu, Huamin Si, Wen Peng, Susheng Song, Wenzhen Liu, Guocheng Hu, Zongxiu Sun, Daoxin Xie, Chuanqing Sun

**Affiliations:** 1 Department of Biological Sciences and Biotechnology, Tsinghua University, Beijing, People's Republic of China; 2 State Key Laboratory of Rice Biology, China National Rice Research Institute, Hangzhou, Zhejiang, People's Republic of China; 3 State Key Laboratory of Plant Physiology and Biochemistry, National Center for the Evaluation of Agricultural Wild Plants (Rice), Laboratory of Crop Heterosis and Utilization of the Ministry of Education, Beijing Key Laboratory of Crop Genetic Improvement, Department of Plant Genetics and Breeding, China Agricultural University, Beijing, People's Republic of China; 4 Chemistry and Life Science College, Tianjin Normal University, Tianjin, People's Republic of China; Gothenburg University, Sweden

## Abstract

Photoperiod and temperature are two important environmental factors that influence the heading-date of rice. Although the influence of the photoperiod on heading has been extensively reported in rice, the molecular mechanism for the temperature control of heading remains unknown. This study reports an early heading mutant derived from tissue culture lines of rice and investigates the heading-date of wild type and mutant in different photoperiod and temperature treatments. The linkage analysis showed that the mutant phenotype cosegregated with the *Hd1* locus. Sequencing analysis found that the mutant contained two insertions and several single-base substitutions that caused a dramatic reduction in *Hd1*mRNA levels compared with wild type. The expression patterns of *Hd1* and *Hd3a* were also analyzed in different photoperiod and temperature conditions, revealing that *Hd1* mRNA levels displayed similar expression patterns for different photoperiod and temperature treatments, with high expression levels at night and reduced levels in the daytime. In addition, *Hd1* displayed a slightly higher expression level under long-day and low temperature conditions. *Hd3a* mRNA was present at a very low level under low temperature conditions regardless of the day-length. This result suggests that suppression of *Hd3a* expression is a principle cause of late heading under low temperature and long-day conditions.

## Introduction

The transition from vegetative to reproductive growth is a critical event in the life cycle of higher plants and is regulated by both endogenous and environmental signals. There are two major environmental factors that influence this transition: photoperiod and temperature. Plants can perceive the change in daylength, or photoperiod, and their response to this change determines whether they will flower or not. Plants can be divided into three groups based on photoperiod flowering response: long-day plants, short-day plants and day-neutral plants [1]–[3]. Rice is a short-day plant that has an early heading-date under short-day (SD) conditions and a delayed heading-date under long-day (LD) conditions.

The pathways controlled by the photoperiodic flowering response were elucidated by different flowering mutants in Arabidopsis [4]. However, QTL analysis of heading-date contributed greatly to our understanding of this pathway in rice because mutants were rarely screened. Dr. Yano's group identified 14 QTLs, *Hd1* to *Hd14*, using an F_2_ population, backcross lines and NIL lines of a cross between the *indica* and *japonica* subspecies of rice [5]–[10]. Recently, several genes involved in photoperiodic flowering have been cloned in rice, such as *SE5*, *Hd1*, *Hd3a*, *Hd6* and *Ehd1*. *SE5* was cloned from a *se5* mutant, which is photoperiod insensitive and displays a dramatically early flowering phenotype. This gene encodes a key hemeoxygenase enzyme involved in phytochrome chromophore biosynthesis; therefore, *se5* mutants are completely deficient in their photoperiod response and in spectrophotometrically detectable phytochromes [11].

The *Hd1* gene is an ortholog of *CO* in Arabidopsis, and encodes a transcription factor with a zinc finger domain. This is a major QTL controlling response to photoperiod and has dual functions in the control of rice heading, serving as a promoter of heading under SD conditions and an inhibitor under LD conditions [12]. The interaction of *SE5* and *Hd1* suggests that the *se5* mutation does not affect the diurnal mRNA expression of *Hd1 (SE1)* upon floral transition. *se1se5* double mutants are more similar to the *se5* single mutant [13]. *Ehd1* encodes a B-type response regulator, and can promote flowering independently of *Hd1* under SD conditions. It has been cloned using a cross-combination between T65 and an accession of African cultivated rice IRGC104038 (*Oryza glaberrima* Steud.). T65 contains two loss-of-function alleles, with both *ehd1* and *hd1* exhibiting a late heading phenotype, whereas *O. glaberrima* IRGC104038 and Nipponbare contain functional *Ehd1* alleles conferring an early heading phenotype [14]. The cloning of *Ehd1* showed that there are at least two independent pathways that promote rice flowering under SD conditions.

*Hd3a* is an ortholog of Arabidopsis *FT*, and encodes a phosphatidylethanolamine-binding protein [15]. This protein can promote the transition of rice flowering and activates flowering under SD conditions. Moreover, the *Hd3a* protein can move from the leaf to the shoot apical meristem (SAM) and induce flowering in rice [16]. The interaction of *Hd1* and *Hd3a* shows that *Hd3a* is regulated by *Hd1*, which is downstream of *Hd1*. *Hd1* can increase *Hd3a* expression to promote heading under SD conditions, but *Hd3a* exhibits very low or no expression under LD conditions [13], 15. Actually, short-day plants mainly detect the length of the night rather than the daylength, using a night-break response [17]. A recent study showed that the mechanism of the night-break response was suppression of *Hd3a* expression [18].

*Hd6* is a QTL involved in photoperiod sensitivity in rice, and encodes the α subunit of the protein kinase CK2 (CK2α). Nipponbare (*Oryza sativa* L. ssp. *japonica*) contains a non-functional allele of *hd6* and exhibits early heading, but Kasalash (*Oryza sativa* L. ssp. *indica*) contains a functional allele of *Hd6* and exhibits late heading [19]. In the photoperiodic control pathway of rice, *OsGI* is located upstream of *Hd1* and *Hd3a*. Overexpression of *OsGI* caused the promotion of *Hd1* mRNA levels and the suppression of *Hd3a* mRNA levels [20]. Moreover, the study showed that *OsMADS51* was a novel flowering promoter that transmitted a SD promotion signal from *OsGI* to *Ehd1* [21].

Temperature is another important environmental factor. Low temperature signals are involved in vernalization pathways and have been extensively studied in Arabidopsis [22]–[26]. Furthermore, Blázquez MA *et al.* [27] and Halliday KJ *et al.* [28] investigated the effect of ambient temperature on flowering time respectively, and found that ambient temperature ultimately affected the expression of the floral pathway integrator *FT*. As is well known, the heading-date of rice is delayed under low temperature conditions, but the molecular mechanism of this pathway is unknown. In contrast to photoperiod, very few studies have been undertaken on heading-date related to temperature responses in rice. This study reports a photoperiod response mutant that displays an early heading phenotype under LD conditions. Linkage analysis shows that this phenotype cosegregates with the *Hd1* locus. The expression levels of *Hd1* and *Hd3a* are analyzed for different photoperiod and temperature treatments to explain the phenomenon of late heading at low temperatures in rice.

## Results

### The phenotype of the mutant

**Figure 1:**
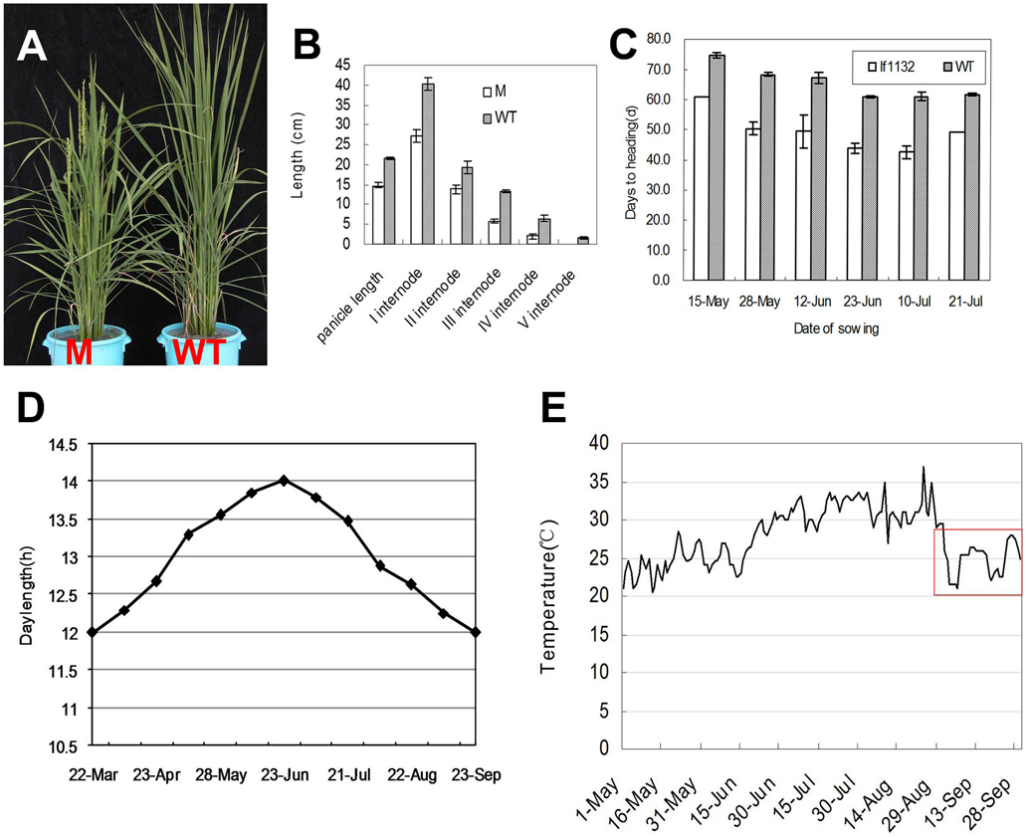
The phenotype of the mutant. A: the phenotype of the mutant and wild type, M is mutant *lf1132*; WT is Zhonghua 11. B: the panicle length and internode length for mutants and wild type. 15 total plants were investigated from five repeats containing three individuals. C: The heading-date of the wild type and mutant on different sowing-dates. Wild type and mutant were planted in the CNRRI experimental field, Zhejiang province on six sowing-dates from 15, May to 21, July 2007. D: The change in photoperiod during different sowing-dates. E: The change in temperature (mean value of everyday temperature) during different sowing-dates. Red box indicates the temperature of the heading period at the last sowing-date, 21, July.

The *lf1132* mutant was derived from a tissue culture line of Zhonghua 11 (*Oryza sativa* L. ssp. *japonica*), and displayed an early heading phenotype in the China National Rice Research Institute's natural field (CNRRI; Fig. 1A). The growth biomass of the mutant was low. For example, its panicle length, plant height and tiller number were all small (Fig. 1B). A backcross was made between *lf1132* and Zhonghua 11 in order to analyze the genetic behavior of the mutant. There were 253 early heading plants (days to heading was 57 d), 513 mid-type but nearly late heading plants (days to heading was 70 d) and 254 late heading plants (days to heading was 76 d) in 1020 F_2_ plants, indicating that this early heading-date phenotype was controlled by a single gene.

To further investigate the heading-date of the mutant, mutant and wild type plants were sowed on different days at regular intervals from May 15 to July 21, 2007 on the CNRRI experimental fields. The weather was recorded each day. The heading-date of the mutant was significantly earlier than the wild type, 14, 18, 17, 16, 18, and 11 days earlier for the different sowing-dates from 15, May to 21, July (Fig. 1C). Days to heading for the mutant and wild type were all shorter for later sowing-dates, except for the last sowing-date, July 21. According to the curves of photoperiodic change, day length shortened gradually with delayed sowing-date (day length increased from 13.3 h to 14 h and then decreased to 12.1 h from the first sowing-date, May 15, to the last heading-date, September 17) (Fig. 1D). Temperature increased gradually with delayed sowing-date except for the last sowing-date. From the last sowing-date July 21 to the last heading-date September 17, the temperature decreased due to continuous rain and cloudy weather in the heading period (The average temperature below 24°C is shown by a red box) (Fig. 1E). Based on the changes in photoperiod and temperature, we speculated that the photoperiod and temperature had effects on the changes in heading-date of wild type and mutant.

### The analysis of heading-date under different photoperiod and temperature conditions

**Figure 2:**
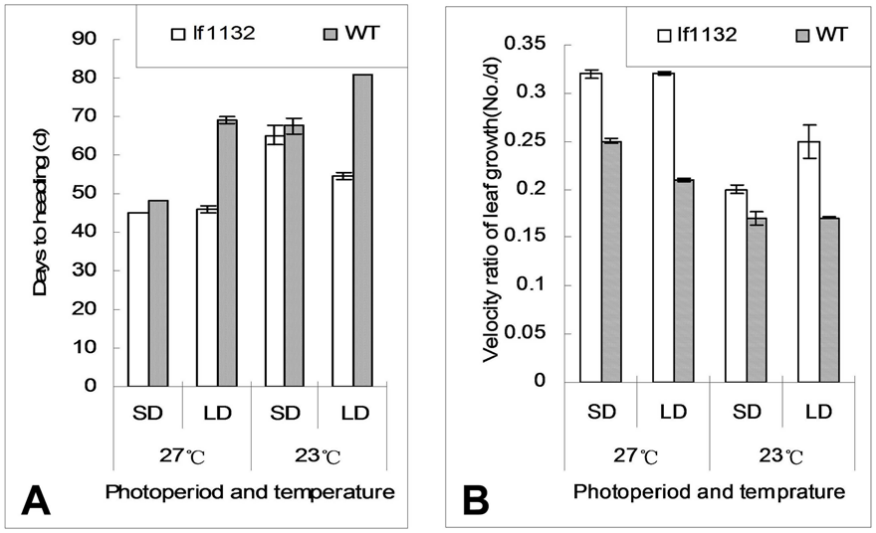
The heading-date of the mutant and wild type for different photoperiod and temperature treatments. *lf1132* and wild type plants were planted in the CNRRI experimental fields, and two week old seedlings were transferred to phytotrons with different photoperiod and temperature treatments. The heading-date for each treatment was observed and recorded for at least 10 plants. Four phytotrons were used: LD, 27°C phytotron; LD, 23°C phytotron; SD, 27°C phytotron; SD, 23°C phytotron; A: the heading-date under different photoperiods and temperatures; B: The velocity ratio of leaf growth (VRL) for the mutant and wild type under different photoperiods (SD and LD) and temperatures (27°C, 23°C). LD treatment: 14.5 h light and 9.5 h dark; SD treatment: 11.5 h light and 12.5 h dark.

To further investigate the effects of photoperiod and temperature on heading-date, mutant and wild type plants were grown in four phytotrons with combinations of different photoperiod treatments (SD: 11.5 h light, 12.5 h dark and LD: 14.5 h light, 9.5 h dark) and temperature treatments (high temperature: 27°C and low temperature: 23°C). Under high temperature conditions, days to heading for the mutant were 45 days and 45.8 days under SD and LD conditions, respectively, but days to heading for the wild type were 48 days and 69 days under the same conditions (Fig. 2A, Table 1), indicating that the mutant had a lower photoperiod sensitivity. The heading-date of the mutant was 3 days earlier than wild type under SD conditions, and about 14 days earlier than wild type under LD conditions. The SD promotion rates for the wild type and mutant plants were 30.4% and 1.7%, respectively, indicating that the SD conditions had a marked promoting effect on wild type, but no obvious effect on the mutant (Table 1).

**Table 1:** The effect of different photoperiods and temperatures on heading-date.

	*lf1132* (Mutant)				Zhonghua 11 (Wild type)			
	SD	LD	Delayed days (d)	SD promotion rate (%)	SD	LD	Delayed days (d)	SD promotion rate (%)
Days to heading in 27°C (d)	45	45.8	0.8	1.7	48	69	21	30.4
Days to heading in 23°C (d)	65.2	54.6	−10.6	−19.4	67.6	81	13.4	16.5
Delayed days (d)	20.2	8.8			19.6	12		
High temperature promotion rate (%)	30.7	16.1			29	14.8		

Notes: Zhonghua 11 and *lf1132* were grown in phytotrons with four different treatments. Heading-date was investigated at least 10 plants for each treatment.

Under low temperature conditions, days to heading for the mutant were 65.2 days and 54.6 days under SD and LD conditions respectively, whereas days to heading for the wild type were 67.6 days and 81 days under the same conditions (Fig 2A, Table 1). The heading-date for the mutant was 2.4 days earlier than for the wild type under SD conditions, and about 27 days earlier than wild type under LD conditions. Interestingly, SD treatments did not promote heading in the mutant; however, the LD treatment promoted heading in the mutant at low temperatures. The SD promotion rate for the wild type was 16.5%, but the SD promotion rate for the mutant was negative, −19.4% (Table 1), indicating that photoperiod had a negative effect on the heading-date of the mutant under low temperature conditions. Moreover, the heading-date of the wild type and mutant were delayed under low temperature conditions regardless of the SD or LD treatment, indicating that a low temperature treatment had an inhibitive effect on the heading-date of rice (Table 1). The high temperature promotion rates of the wild type and mutant under SD conditions were 29% and 30.7%, respectively, and 14.8% and 16.1% under LD conditions, respectively (Table 1). The high temperature promotion rates under SD conditions were higher than those under LD conditions, suggesting that temperature positively regulated heading-date in rice.

Usually, the number of leaves can be used to measure the time of plant flowering before the first flower is produced. To analyze heading-date in detail, we investigated the main-stem leaf number (LN) of the mutant and wild type under different photoperiodic and temperature treatments. At high temperature conditions, the LNs of the mutant were all 9 leaves for the SD and LD conditions, but the LNs of the wild type were 8 and 11 leaves under SD and LD conditions, respectively (Table 2). Photoperiod did not affect the LNs of the mutant, but the LNs of the wild type increased from SD to LD under high temperature conditions. The velocity ratio of leaf growth (VRL) of the mutant was higher than that of wild type, whether under SD or LD conditions (Fig. 2B), indicating that the development of the mutant was faster than that of the wild type. Under low temperature conditions, the LNs of the mutant were 10 and 9 leaves under SD and LD conditions respectively, rather than 9 and 11 leaves under SD and LD conditions, respectively, for wild type (Table 2). Low temperature conditions reduced VRLs for both SD and LD conditions, suggesting that low temperature inhibited the growth and development of rice. Furthermore, LNs decreased under LD and low temperature treatments in the mutant, in contrast to the wild type LNs, which increased with the change of day length from SD to LD, whether under the high temperature or low temperature treatments, indicating that LD conditions promoted the heading-date of the mutant under low temperature treatment. Correspondingly, the heading-date of the mutant was about 11 days earlier from SD to LD conditions under the low temperature treatment; however, the wild type heading-date was delayed under LD conditions regardless of temperature (Table 1). The results of the LNs were in agreement with the results of the heading-dates above.

**Table 2:** Leaf number of mutant and wild type in different photoperiod treatments and temperature treatments.

	27°C		23°C	
	SD	LD	SD	LD
Mutant	9	9	10	9
Wild type	8	11	9	11

Notes: Zhonghua 11(wild type) and *lf1132* (mutant) were grown in phytotrons with four different treatments. The main-stem leaf number was investigated at least 10 plants for each treatment in four phytotrons.

### Sequence analysis of the Hd1 locus in lf1132 and wild type

**Figure 3:**
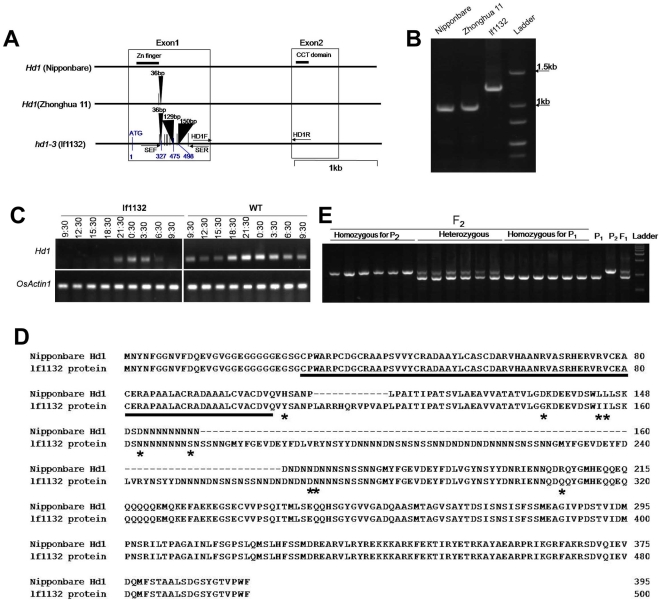
Sequence analysis of *Hd1* in *lf1132* and wild type. A: The sequence differences between wild type and *lf1132*. The black triangle represents insertion; vertical lines represent single-base substitutions; blue vertical lines and numbers are relative positions in *hd1-3*. SEF and SER shown by arrows are primers to detect the 315 bp insertion. B: PCR detection of the 315 bp insertion on the *hd1-3* locus for *lf1132*. C: The expression of *Hd1* in the wild type and mutant. Leaves were harvested from 30 day old seedlings at the indicated times (once every 3 h for 24 h) in natural fields (day-length is about 14 h light and 10 h dark) and RT-PCR was carried out for the analysis of *Hd1* expression. Primer pairs HD1F and HD1R were used for the analysis of *Hd1* expression in RT-PCR. D: Deduced amino acid sequence of the Hd1 and deduced lf1132 proteins. The black line indicates the zinc-finger domain; asterisks are amino acid substitutions between the Nipponbare Hd1 protein and the deduced lf1132 protein. E: the linkage analysis of the mutant and *Hd1* locus. P_1_ is Zhonghua 11; P_2_ is *lf1132*.

In previous studies, the *se1* mutants HS66 and HS110 both displayed an early heading phenotype with lower photoperiod sensitivity, and Yano *et al.* [12] later confirmed that *Se1* was an allele of *Hd1*. Sequence analysis found that HS66 and HS110 contained a 43 bp deletion and a 433 bp insertion, respectively [12]. In addition, HS66, HS110 and their progenitor variety Ginbouzu all contained a 36 bp insertion and a nucleotide substitution compared to Nipponbare. As the phenotype of *lf1132* was similar to the phenotype of *se1* mutants, we investigated whether *lf1132* harbored a mutation of the *Hd1* locus through sequencing verification of *lf1132* and Zhonghua 11. We found that the *Hd1* locus contained a 36 bp insertion and a nucleotide substitution compared to Nipponbare in Zhonghua 11 (Fig. 3A). However, the *Hd1* locus contained 6 new single-base substitutions and two new insertions, a 129 bp insertion and a 150 bp insertion in *lf1132*, in addition to a 36 bp insertion and a nucleotide substitution at the same position compared to Zhonghua 11 (Fig. 3A). These two new insertions are located in the first exon and consist of genome duplications with several single-base substitutions. Three insertions (36 bp insertion, 129 bp insertion and 150 bp insertion) are located at the position 327 bp, 475 bp and 498 bp far from ATG site, respectively. The distances between two near insertions are 148 bp and 23 bp respectively. Therefore, these two new insertions resulted in reading-frame shift in mutated region (from the position 327 bp to 498 bp), but no reading-frame shift after the position 499 bp. These insertions and single-base substitutions do not locate in the zinc-finger domain and CCT domain, also do not cause a premature stop codon in *lf1132* (Fig. 3A). We speculated that the early heading phenotype was caused by 6 new single-base substitutions and two new insertions, but not by the 36 bp insertion and substitution because Zhonghua 11 also contained these same elements. SEF and SER primers can successfully detect these two new insertions in *lf1132*, but not in Zhonghua 11 or Nipponbare (Fig. 3B). These results indicate that this gene is an allele of *Hd1*, and is designated *Hd1-3*.

To investigate the expression level of *hd1-3* in *lf1132*, RNAs were isolated from 30 day seedlings in the natural field at the indicated times (from 9:30 am to 9:30 am on the next day, one sample every 3 h). RT-PCR showed that *hd1-3* mRNA in *lf1132* was severely reduced compared to wild type (Fig. 3C). Although *lf1132* contains two new insertions and 6 new single substitutions, it can still be translated to produce a protein since these mutations do not cause a premature stop codon (Fig. 3D). In addition, several amino acids are changed from the Hd1 to deduced lf1132 protein, and these mutations do not occur within the zinc-finger domain and CCT domain (Fig. 3D). These mutations may only affect Hd1 protein activity; therefore, *hd1-3* is still expressed at a lower level, which indicates that the *hd1-3* allele in *lf1132* seems to be a knock-down of function allele (Fig. 3C).

To support the idea that the phenotype of *lf1132* was caused by mutation of the *Hd1* locus, we produced an F_2_ population by crossing with *lf1132* and Zhonghua 11. Of the total 1020 F_2_ plants, 253 plants displayed an early heading phenotype. We sampled the leaves of 253 early heading plants and randomly sampled 96 late heading plants (containing 64 mid-type heading plants and 32 late heading plants) to isolate genomic DNA. PCR detection showed that all 253 early heading plants displayed bands of *lf1132*. 31 plants displayed the bands of Zhonghua 11 and 65 plants displayed heterozygous bands in 96 late heading phenotype plants (Fig. 3E). This result suggests that the phenotype of *lf1132* is caused by the *hd1-3* locus.

### Analysis of the expression of Hd1 and Hd3a under different photoperiod and temperature treatments

**Figure 4:**
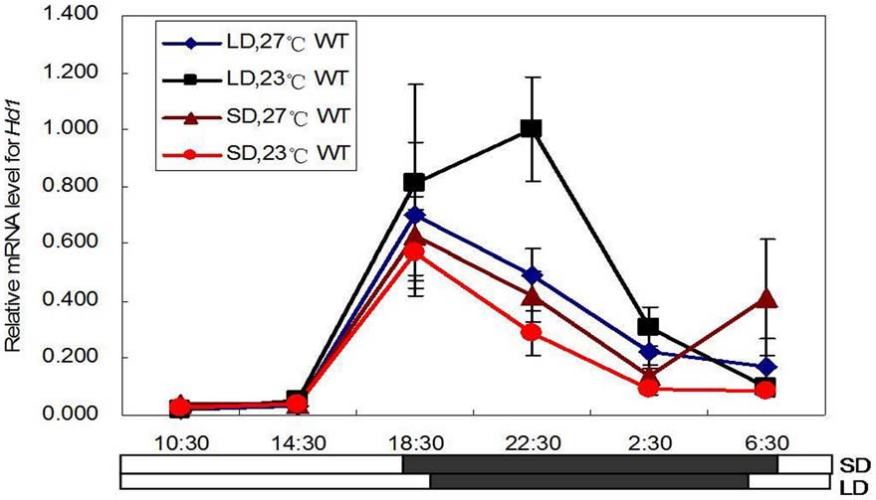
*Hd1* expression under different photoperiods and temperatures. Leaves were harvested from 33 day old plants at the indicated times (once every 4 h for 24 h) in phytotrons, and real-time PCR was carried out for analysis of *Hd1*. M is *lf1132*; WT is Zhonghua 11.

To reveal the molecular mechanism of heading-date in rice, we used real-time PCR to analyze the expression levels of *Hd1* in wild type plants under different photoperiod and temperature conditions. *Hd1* mRNA levels displayed similar expression patterns in different photoperiod and temperature treatments, which had high expression levels during the night and exhibited a peak at the onset of darkness (Fig. 4). *Hd1* mRNA levels were reduced in the daytime, indicating a night-time expression pattern, consistent with previous observations in rice [13] and similar to *CO* expression in Arabidopsis [29]. In addition, *Hd1* mRNA levels displayed slightly higher expression under LD and low temperature conditions. However, in general, *Hd1* mRNA levels were largely unaffected by photoperiod and temperature, and displayed a constitutive expression pattern under different photoperiod and temperature conditions (Fig. 4). The expression of Arabidopsis *CO* [30], an ortholog of *Hd1*, was also largely unaffected by ambient temperature [27], which is similar to our result. Together with our data of delayed heading under LD conditions and low temperature treatments in wild type (Table 1) and previous studies [12], [13], this result suggests that *Hd1* may play an inhibitory function under LD and low temperature conditions.

**Figure 5:**
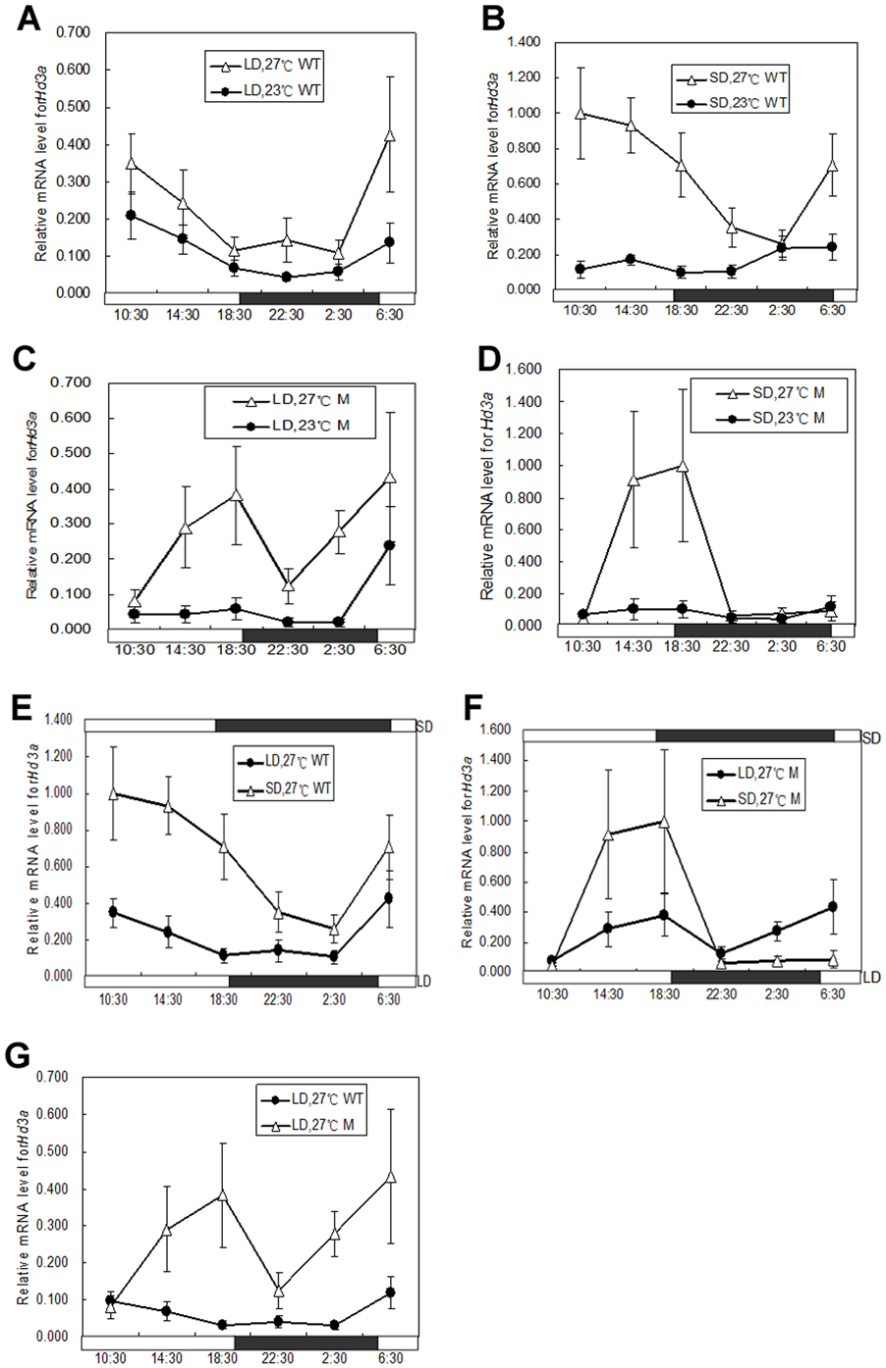
*Hd3a* expression under different photoperiods and temperatures. Leaves were harvested from 33 day old plants at the indicated times (once every 4 h for 24 h) in phytotrons, and real-time PCR was carried out for the analysis of *Hd3a* expression. M is *lf1132*; WT is Zhonghua 11. A, B, C, D are the *Hd3a* expression profiles under high temperature and low temperature; A: wild type under LD condition; B: wild type under SD condition; C: mutant under LD condition; D: mutant under SD condition. E and F are the *Hd3a* expression profiles for the wild type and mutant under different photoperiods at high temperature; E: wild type; F: mutant. G presents the *Hd3a* expression profile of the mutant and wild type under LD conditions.

Previous results have shown that *Hd3a* was an activator of rice flowering and was regulated by *Hd1*. Higher mRNA levels for *Hd3a* under SD conditions promoted the heading of rice, whereas lower *Hd3a* mRNA levels under LD conditions inhibited the heading of rice [13], [15]. Therefore, we analyzed the *Hd3a* mRNA levels in wild type and mutant plants under different photoperiod and temperature treatments. The results showed that *Hd3a* mRNA was present at very low levels under low temperature conditions both in the wild type and the mutant, regardless of the SD or LD condition (Fig. 5A, B, C, D). Together with the results of delayed heading in low temperature treatments (Table 1), this indicates that late heading under low temperature conditions was caused by the suppression of *Hd3a* expression. On the other hand, *Hd3a* mRNA exhibited a diurnal expression pattern, with high expression occurring during the daytime. *Hd3a* mRNA displayed higher expression levels under SD conditions, with lower expression levels under LD conditions (Fig. 5E, F). Moreover, the expression curve and the timing of the peaks were different for the wild type and mutant under SD conditions. *Hd3a* mRNA levels increased beginning at dawn with a peak at the onset of light in wild type, but in the mutant, they increased from the onset of light and had a peak at the onset of dark in the mutant (Fig. 5E, F), indicating that *Hd1* regulates *Hd3a*. In addition, *Hd3a* mRNA levels in the mutants were significantly higher than those in the wild type under LD conditions (Fig. 5G), suggesting that *Hd1* negatively regulates *Hd3a* under LD conditions.

## Discussion

### The diversity of Hd1 alleles in different rice varieties

Several *Hd1* alleles have been reported so far, and they display sequence diversity. Nipponbare contained a functional allele of *Hd1* with a zinc finger domain and a CCT domain, and Kasalath contained a knockdown of function allele, *hd1*, with lots of single-base substitutions, deletions and insertions compared to *Hd1* of Nipponbare [12]. HS66 and HS110 also contained a knockdown of function allele, *se1*, with a 43 bp deletion and a 433 bp insertion, respectively [12]. Doi *et al.* [14] reported the loss-of-function allele *hd1* with a 1901 bp insertion in the nearby CCT domain in T65. Takahashi *et al.* [31] analyzed the diversity of *Hd1* alleles using a core collection of 64 rice cultivars and found that *Hd1* loci have very high polymorphisms. The *Hd1* alleles in the core collection were grouped into 17 types, and 15 distinct proteins [31]. Here, we report a new allele of *Hd1*, named *hd1-3*, with two insertions in the first exon. This allele caused a dramatic reduction of *Hd1* mRNA and displayed an early heading phenotype under SD and LD conditions, especially under LD conditions. Understanding the diversity of these alleles can contribute to our understanding of *Hd1* function. In Arabidopsis, the *CO* gene can promote flowering under LD conditions but exerts no phenotypic change on flowering time under SD conditions [30]. However, *Hd1*, a homolog of *CO*, showed a dual function, promoting heading under SD conditions but inhibiting heading under LD conditions [12], [13]. In this study, the heading-date for mutant was only 3 days earlier than wild type under SD conditions, but 14 days earlier than wild type under LD conditions. Furthermore, *Hd1* exhibits a dramatically inhibited function under LD conditions, but these results do not show a promoting function of *Hd1* under SD conditions.

### The interaction of photoperiod and temperature in the pathway of rice flowering

The heading-date of rice is regulated by both photoperiod and temperature signals. Regulation by photoperiod has been extensively studied using the natural genetic variation in the species. However, the molecular mechanism of heading-date control by temperature remains unknown. In Arabidopsis, temperature can regulate flowering time by the vernalization pathway. Vernalization is important for winter plants, and it can regulate flowering time through the suppression or promotion of *FLC* expression [22], [24]–[26]. Unlike Arabidopsis, rice is a summer and short-day plant that can flower without passing through a vernalization stage. The photoperiod response in rice is contrary to Arabidopsis. So far, the homologous genes to *FLC* have still not been found in rice; and it remains unknown whether other genes substitute for *FLC* in rice.

In this study, the heading-date of rice was delayed in low temperature conditions due to the suppression of *Hd3a* expression under low temperature conditions. Moreover, the heading-date of mutant under low temperature was 11 days earlier under LD conditions than under SD conditions. This is an abnormal phenomenon in rice because rice is a short-day plant and its heading-date is inhibited under LD conditions and promoted under SD conditions. We analyzed the *Hd1* and *Hd3a* expression profiles and could not explain this result. On the one hand, deduced lf1132 protein contained 93 amino acids insertion and several substitutions of amino acids, which suggested that the function of lf1132 protein may be altered. On the other hand, Post-translational regulation of Hd1 protein likely plays a crucial role in the regulation of rice heading-date. It has been reported that post-translational regulation of CO protein plays an important role in Arabidopsis. Photoreceptors such as *PHYA*, *PHYB* and *CRY2* can affect the stability of CO protein to regulate flowering time. *PHYA* and *CRY2* can increase the accumulation of CO and stabilize CO to promote flowering in far-red or blue light conditions, and *PHYB* can reduce the accumulation of CO to delay flowering in red light condition. The accumulation of CO reduced when mutations occurred in *PHYA* and *CRY2*, whereas the accumulation of CO increased when mutations occurred in *PHYB* [32]–[34]. Recently, Jang *et al.* reported that *COP1* can promote the degradation of CO protein mainly in the dark; however, the degradation of CO protein in the morning is independent of *COP1* by a phytochrome B-dependent mechanism [32]. Also, Liu *et al.* reported that *COP1* inhibits flowering by promoting the ubiquitin-mediated proteolysis of CO in darkness, and CRY-mediated signal may negatively regulate *COP1* to stabilize CO protein [33]. In addition, Laubinger *et al.* reported that *spa1 spa3 spa4* mutants exhibit strongly increased CO protein levels, which are not caused by a change in *CO* gene. Their further experiments showed that SPA proteins can interact with CO protein in vivo through CCT-domain of CO to regulate flowering time by controlling the stability of CO protein [35].

Futhermore, *Hd3a* mRNA level reduced dramatically in rice under low temperature in our study. We speculated that there might be one pathway regulating rice heading-date based on temperature-dependent signals. In Arabidopsis, several late-flowering mutants displayed a temperature-dependent phenotype such as *fha*, *fca* and *fve* [27]. The *fha* mutant is very sensitive to ambient temperature and displays a dramatically delayed phenotype under low temperature conditions. Further experiments confirmed that the *fha* delayed phenotype at 16°C was caused by the far lower activity of *PHYA* at low temperatures [27], [28]. *fca* and *fve* mutants are insensitive to ambient temperature and flower at the same time under different temperature conditions (23°C and 16°C). Further experiments suggested that the temperature response of *fca* and *fve* was mediated by *FLC*, and that it ultimately caused the dramatic reduction of *FT* expression [27], [28]. *FT* is an integrator of the floral inductive and a homolog of *Hd3a* in rice. In our studies, *Hd3a* expression was reduced dramatically under low temperature conditions for both LD and SD in the mutant and wild type (Fig. 5A, B, C, D). This result is consistent with *FT* expression under low temperature conditions in Arabidopsis.

In addition, we speculate that there might be a pathway of interaction between photoperiod and temperature signals in rice. That is, the genes involved in the temperature and photoperiod signaling pathways might affect the heading-date of rice by interaction with each other. Also, the genes involved in the photoperiod pathway might be closely linked to the locus of temperature response. Nakanawa *et al.* [36] analyzed the flowering response of rice to photoperiod and temperature by QTL analysis, and found that *Hd1* and *Hd2* responded to both photoperiod and temperature. Moreover, Lin et al. [6] identified the *Hd9* locus and proposed a hypothesis: *Hd9* is involved in characteristics other than photoperiod sensitivity, such as high temperature treatment. Later, Nakanawa's result indicated that *Hd9* was involved in thermal response and supported this hypothesis [36]. These results suggest that these QTLs related to photoperiod and temperature may either be independent of or dependent on each other. In this study, the wild type was sensitive to both photoperiod and temperature; however, the mutant was insensitive to photoperiod but sensitive to temperature. Therefore, the days to heading for the wild type displayed a regular shortening trend because photoperiod and temperature play a shared role in the heading-date of the wild type, but displayed an irregular trend in the mutant because only temperature plays a role in heading-date (Fig. 1C). This result indicates that temperature has a strong effect on photoperiod-insensitive cultivars, and might confirm the above hypothesis that the response of photoperiod depends on temperature. Beyond all doubt, temperature has an important influence on rice heading-date, especially under low temperature conditions. Our results demonstrate that *Hd3a* mRNA levels were dramatically reduced under low temperature conditions for both SD and LD conditions. This indicates that temperature and photoperiod can both regulate the heading-date of rice by controlling *Hd3a* mRNA levels. However, it still remains to be determined how temperature regulates *Hd3a* expression. Further studies may reveal the mechanism of heading-date control by photoperiod and temperature.

## Materials and Methods

### Plant materials

The rice varieties Zhonghua 11 (*Oryza sativa* L. ssp. *japonica*) and *lf1132* (mutant) were planted in the experimental field of the CNRRI, Hongzhou, Zhejiang province (N30°05′, E119°05′). *lf1132* was derived from a tissue culture line of Zhonghua 11. About 150 Zhonghua 11 and *lf1132* plants each were sowed on May 15, May 28, June 12, June 23, July 10 and July 21, 2007. Heading-date was measured according to the 50% heading-rate of the population. F_1_ plants derived from crosses of *lf1132* and Zhonghua 11 were grown in Sanya, Hainan province (N18°15′, E109°43′) and F_2_ plants were grown in Beijing (N 39°48′, E116°28′).

### Plant growth condition in phytotrons

Four treatments were conducted in phytotrons (KOITOTRON S-153W-Special), with two photoperiods and two temperatures: LD, 27°C; LD, 23°C; SD, 27°C; SD, 23°C. The day length parameters are: LD, 14.5 h light and 9.5 h dark; SD, 11.5 h light and 12.5 h dark. The high temperature treatment is 27°C (the weighted average from 24°C to 30°C), and the low temperature treatment is 23°C (the weighted average from 20°C to 26°C). Zhonghua 11 and *lf1132* were sowed in natural fields, and two-week old seedlings were transferred to phytotrons. Heading-date and leaf numbers were investigated for at least 10 plants for each treatment. The SD promotion rate (%) and the high temperature promotion rate (%) were calculated by the following formulas:

The SD promotion rate (%) = (Days to heading in LD−Days to heading in SD)/Days to heading in LD×100%

The high temperature promotion rate (%) = (Days to heading in low temperature−Days to heading in high temperature)/Days to heading in low temperature×100%

### The linkage analysis of the mutant and the Hd1 locus

About 1020 F_2_ plants derived from the cross between *lf1132* and Zhonghua 11 were planted, and their phenotypes were identified according to heading-date. The leaves were used to extract genomic DNA, and PCR was carried out for linkage analysis. The primers were: SEF: 5′-AGAGGAACAGGAGAAGACGC-3′ and SER: 5′-ACCACTATGCTGCTGCTCAC-3′. The amplification conditions were as follows: 1 min at 95°C; 30 cycles of 30 sec at 94°C, 30 sec at 58°C, and 1 min at 72°C followed by 5 min at 72°C. For analysis of expression in the mutant and wild type, RNAs were isolated from 30 day old seedling leaves in natural fields using Trizol solution (Invitrogen, USA) and treated with DNase I (Invitrogen, USA). The cDNAs were synthesized from 1 µg of total RNA using M-MLV reverse transcriptase (TaKaRa, Dalian, China). One microliter of cDNA was used for RT-PCR analysis with gene specific primers. The sequences of the primers were as follows: HD1F:5′- GGTTATGGAGTTGTGGGAGCAGAC-3′ and HD1R:5′- AGTGAAGGGACATCTGAAGCGAGG -3′. *OsActin1* was used as an internal control. The sequences of the primers were as follows: ActinF: 5′-GACTCTGGTGATGGTGTCAGC-3′ and ActinR: 5′-GGCTGGAAGAGGACCTCAGG-3′. The amplification conditions were as follows: 3 min at 94°C; 35 cycles of 30 sec at 94°C, 30 sec at 60°C, and 30 sec at 72°C; followed by 5 min at 72°C for *Hd1*. 3 min at 94°C; 26 cycles of 30 sec at 94°C, 30 sec at 60°C, and 30 sec at 72°C; followed by 5 min at 72°C for *OsActin1*.

### The analysis of the expression of Hd1 and Hd3a

Leaves were harvested from 33 day old plants in phytotrons at the indicated times, and total RNAs were extracted using Trizol solution (Invitrogen, USA) and treated with DNase I (Invitrogen, USA). The cDNAs were synthesized from 1 µg of total RNA. One microliter of cDNA was used for real-time PCR analysis of gene expression performed with SYBR Green PCR master mix (Tiangen, Beijing, China) and the gene specific primers, and real-time PCR was performed in a Stratagene Mx3000P™ Thermal System (STRATAGENE, USA). The primer sequences were as follows: HD1F and HD1R were the same as above for the *Hd1* gene; HD3aF: 5′-TTGGTAGGGTTGTGGGTGATGTGC-3′and HD3aR: 5′-AGGTTAGGGTCACTTGGGCTTGGT-3′ for the *Hd3a* gene. Data were analyzed using the Mx3000P sequence detection system in accordance with the instruction manual. The 2^-ΔΔ*C*t^ Method described by Livak KJ was used for the analysis of relative gene expression [37]. Three replicates of each reaction were performed, and *OsActin1* was used as an internal control to relatively quantify the target gene expression. The amplification conditions were as follows: 2 min at 95°C; 40 cycles of 20 sec at 95°C, 30 sec at 60°C, and 30 sec at 68°C.
